# Diagnostics in anaemia of chronic disease in general practice: a real-world retrospective cohort study

**DOI:** 10.3399/bjgpopen18X101597

**Published:** 2018-07-25

**Authors:** Annemarie Schop, Karlijn Stouten, Ron van Houten, Jürgen Riedl, Joost van Rosmalen, Patrick JE Bindels, Mark-David Levin

**Affiliations:** 1 PhD Student, Department of Internal Medicine, Albert Schweitzer Hospital, Dordrecht, The Netherlands; 2 Clinical Chemist Resident, Department of Clinical Chemistry, Albert Schweitzer Hospital, Dordrecht, The Netherlands; 3 GP, General Medical Practice Van Houten, Hendrik-Ido-Ambacht, The Netherlands; 4 Clinical Chemist, Department of Clinical Chemistry, Albert Schweitzer Hospital, Dordrecht, The Netherlands; 5 Assistant Professor of Biostatistics, Department of Biostatistics, Erasmus MC, Rotterdam, The Netherlands; 6 Professor in General Practice, Department of General Practice, Erasmus MC, Rotterdam, The Netherlands; 7 Internist-Hematologist, Department of Internal Medicine, Albert Schweitzer Hospital, Dordrecht, The Netherlands

**Keywords:** anaemia of chronic disease, inflammation anaemia, general practice, primary care, diagnosis

## Abstract

**Background:**

Limited research has been performed that focused on the diagnosis of the underlying cause of anaemia of chronic disease (ACD) in general practice or on prevalence data of the underlying causes of ACD in general practice, although this is one of the most common types of anaemia.

**Aim:**

To clarify the diagnostic strategies of GPs in patients newly diagnosed with ACD and to determine the most common underlying causes.

**Design & setting:**

Retrospective cohort study.

**Method:**

Patients newly diagnosed with ACD were selected based on laboratory criteria. ACD was defined as confirmed anaemia and ferritin levels above 100 μg/l combined with decreased iron and/or reduced transferrin. Additional medical information on patients was obtained from the electronic medical files of the GP and/or the referral hospital.

**Results:**

Of the 267 analysed patients with ACD, additional investigations were performed in 205 patients (77%); in 31 patients (12%) the cause was apparent at the time of diagnosis, and for 31 patients (12%) no additional investigations were requested. In 210 (79%) of the 267 patients, an underlying cause was established, with infection (*n* = 68, 32%), autoimmune disease (*n* = 51, 24%) and malignancy (*n* = 48, 23%) as the most frequently observed etiologies. In 35 (13%) of the ACD patients, oral iron supplementation was prescribed by the GP. This was mainly done in patients with severe anaemia or less enhanced ferritin levels.

**Conclusion:**

For most patients with newly diagnosed ACD, the GP undertakes additional investigations to establish underlying causes. However, the cause of ACD remains unknown in a small proportion of patients. The use of oral iron supplementation in these patients requires caution.

## How this fits in

This study provides an overview of the most commonly requested additional investigations by GPs and presents the prevalence of the most common underlying causes of ACD in general practice. ACD is often observed in general practice. Establishing the underlying cause of ACD is essential, since this condition can lead to reduced quality of life and/or increased risks related to possibly detrimental underlying diseases. Finally, this study demonstrates that 13% of ACD patients still receive oral iron supplementation, mainly those patients with severe anaemia and less enhanced ferritin levels.

## Introduction

Anaemia is a common finding in general practice and is associated with increased mortality, physical and cognitive decline, collapse, fractures, frailty, cardiovascular events, and reduced quality of life.^^1,2^^ACD is one of the most common types of anaemia.^^3–6^^ This type of anaemia can be caused by a variety of conditions, including acute and chronic infections, chronic diseases, autoimmune disorders, acute trauma, surgical interventions, renal failure, heart failure, and malignancies.^^7–13^^

ACD is described as a functional iron deficiency caused by elevated hepcidin levels, which implies that oral iron supplementation is unnecessary.^14–16^ Patients with ACD aged ≥50 years have a relative risk for mortality of 1.48 compared to adults without anaemia.^17^ To ensure proper treatment of ACD, the underlying cause needs to be elucidated and treated. If the cause is not clear, additional investigations are required.^2,5,15,16,18,19^ These steps require active participation of the GPs involved with patients with ACD in general practice.

The National Institute for Health and Care Excellence (NICE) published guidelines on the management and treatment of anaemia in patients with chronic kidney disease, which is an underlying cause often observed in ACD patients.^20^ Besides these guidelines, there is not much research available focusing specifically on the diagnosis of the underlying cause of ACD in general practice, or on prevalence data of the underlying causes of ACD in general practice. Therefore, this study aims to clarify the diagnostic strategies employed by GPs for their patients with ACD and provides an overview of the underlying causes as established by GPs and/or medical specialists.

## Method

### Patient inclusion

Patients were recruited as part of a transmural project aimed at anaemic patients aged ≥50 years. The main goal of the project is to improve the care for anaemic patients, and it has been running since 2007.^21^

In patients presenting to one of the 63 participating GPs with symptoms indicative of anaemia, an extensive laboratory protocol was performed that consisted of haemoglobin, mean corpuscular volume, C-reactive protein and/or erythrocyte sedimentation rate, vitamin B12, creatinine, ferritin, folic acid, lactate dehydrogenase, transferrin, reticulocytes, leukocytes, thrombocytes, and serum iron.^18^ Anaemia was defined as haemoglobin ≤13.7 g/dL (8.5 mmol/L) for males and ≤12.1 g/dL (7.5 mmol/L) for females. ACD was defined as confirmed anaemia and ferritin ≥100 μg/l combined with decreased serum iron (<14 μmol/L for males and <10 μmol/L for females) and/or reduced transferrin (below the lower limit of the reference interval).^21,22^ As part of the transmural project, a comment was added to the laboratory results alerting GPs to the most likely cause of anaemia.

For the present study, patients diagnosed with ACD were retrospectively selected from the large database that was compiled between 1 February 2007 and 1 February 2013.

### Data collection

**Figure 1.**
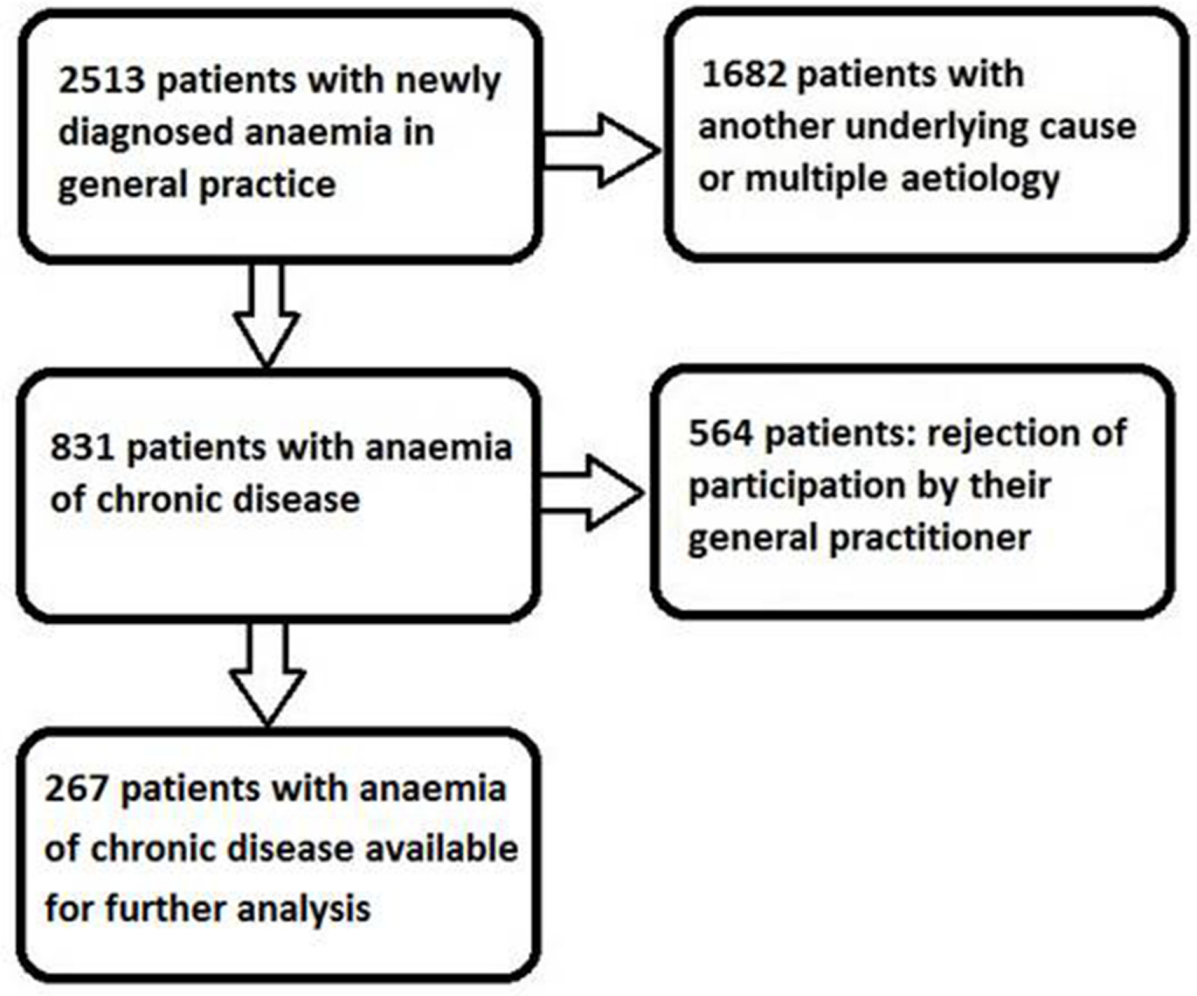
Flow diagram of the selection of study patients.

Clinical data was extracted from the electronic medical files of the GPs and referral hospital. Data could only be collected on the basis of the willingness of the GP to participate in the study and the availability of the data in electronic documentation. If these conditions could not be met, the patient was excluded from the study (*n* = 564) (Figure 1).

Since patients were recruited over a 6-year period, the length of follow-up varied considerably. The end of follow-up was recorded as the date of data collection, or the date of the last available documented data (due to patients changing their GP in case of relocation or migration). If the patient had died before the moment of data collection, the date of death was recorded as the end of follow-up.

Collected clinical information consisted of all diagnostic investigations ordered by GPs, including those requested in consultation with a medical specialist, from the moment of discovery of ACD until the diagnosis of the underlying cause or the first outpatient visit to the referral hospital. These investigations were defined as ‘additional investigations’. In addition, all underlying causes of ACD (those diagnosed by the GP and those diagnosed by the referral hospital's medical specialist) were registered. If the patient was known to suffer from diabetes, chronic lung disease, or heart failure, and no other underlying cause of ACD could be established, these diseases were assigned as the underlying cause of ACD.^7–13^ Any prescription of oral iron supplementation, as well as the time (in weeks) from the confirmation of ACD to the establishment of the underlying cause were also recorded.

### Definitions

Guidelines published by the Dutch College of General Practitioners in 2003 (revised in 2014) recommend handling newly diagnosed ACD in general practice as follows:

1. further diagnostic investigations to clarify the underlying illness (unless an underlying cause is already known); and
2. no oral iron supplementation.^16^

In the present study this was defined as ‘current recommendations’.

### Statistical analysis

The study population was characterised by standard descriptive statistics. Comparisons of the included and excluded patients were performed using the χ^2^ test and independent samples *t*-test (as appropriate). Differences in mean haemoglobin levels between patients referred and not referred to a medical specialist after establishment of ACD were tested using an independent samples *t*-test. Factors associated with the prescription of oral iron supplementation were analysed using the χ^2^ test, independent samples *t*-test, and multivariable logistic regression analysis. The independent variables in the logistic regression were age, sex, haemoglobin, and ferritin. Haemoglobin and ferritin were dichotomised according to the median of those variables. In addition, the same univariable and multivariable analyses were used to investigate factors associated with the recommended diagnostic and therapeutic strategy of patients with ACD.

A two-sided *P*-value ≤0.05 was considered statistically significant. Data were analysed using the SPSS for Windows (version 24).

## Results

### Patient characteristics

Between 1 February 2007 and 1 February 2013, patients with newly diagnosed anaemia were included in a large transmural project.^21^ In 831 (33%) of these 2513 patients, the anaemia was defined as ACD based on predefined laboratory criteria and evaluated by experienced clinical chemists. All tests were performed in the clinical chemistry laboratory of the Albert Schweitzer Hospital.

Because of lack of consent of the GP due to time restraints and loss of follow-up, a total of 267 patients (32%) with newly diagnosed ACD were finally available for the analysis (Figure 1). Characteristics of the study group are presented in Table 1.

**Table 1.** Characteristics of the study population (*n* = 267).

	Mean ±SD	Reference value
Sex	148 male 119 female	
Age, years	74.3 ±10.5	
Male	72.3 ±10.7	
Female	76.7 ±9.7	
**Haemoglobin, g/dl**	11.8 ±1.3	
Male	12.4 ±1.1	13.7–17.7
Female	11.0 ±1.0	12.1–16.1
**Transferrin, g/l**	1.9 ±0.4	2.0–3.6
**Ferritin, µg/l**		
Male	445 ±325	25–250
Female	379 ±373	20–150
**Serum iron, µmol/l**		
Male	7.1 ±3.7	14–28
Female	5.9 ±3.4	10–25

No significant difference was found between the included patients and the non-participants regarding sex (*P* = 0.11), but a small difference in age was found (mean age of included patients 74.3 years versus 73.5 years in non-participants, *P* = 0.05). For both males and females, no significant differences in haemoglobin, ferritin, or transferrin levels were found between the included patients and non-participants (data not shown). In addition, the included patients demonstrated a significantly lower serum iron level compared with the non-participants (*P* = 0.003 for males, *P* = 0.01 for females).

### Additional investigations by GPs

In 205 (77%) of the 267 included patients with newly established ACD, additional investigations were requested by GPs to clarify the underlying illness (Table 2). Moreover, in 78 patients multiple investigations were performed, resulting in a total of 311 investigations. The most frequently requested additional investigations were chest X-ray (24%), referral to an internist (21%), and referral to the emergency room (10%). The cause was already apparent at the time of diagnosing the ACD in 31 patients (12%), mostly due to diabetes mellitus (39%) and autoimmune diseases (32%). For 31 patients (12%), no additional investigations were requested, even though the cause of ACD was not clear. Patients who were referred to a medical specialist (*n* = 145, 54%), either at initial diagnosis or after additional investigations by the GP, had a mean haemoglobin level of 11.7 (range 8.1–13.5) g/dl compared with 11.8 (range 7.9–13.5) g/dl in patients who were not referred (*P* = 0.378).

**Table 2.** Data on established cause and recorded investigations

	Frequency, *n* (%)
Physical examination^a^	17 (5.5)
**X-ray**	
Chest	75 (24.1)
Abdomen	11 (3.5)
Joint	9 (2.9)
Sinus	2 (0.6)
**Ultrasound, abdomen**	28 (9.0)
**CT scan, abdomen/thorax^b^**	12 (3.9)
**Endoscopy**	
Full endoscopy	2 (0.6)
Gastroscopy	5 (1.6)
Colonoscopy	5 (1.6)
**Referral**	
Internist	66 (21.2)
Emergency room	32 (10.3)
Pulmonologist	12 (3.9)
Geriatrician	11 (3.5)
Rheumatologist	8 (2.6)
Other	16 (5.1)
Total	311

^a^ Only physical examinations directly related to a diagnosis of the underlying cause were noted. ^b^ CT scan was always requested in consultation with a medical specialist. CT= computed tomography.

### Underlying causes of ACD

In 210 (79%) patients an underlying cause of ACD was determined, most frequently infection (*n* = 68, 32%), autoimmune disease (*n* = 51, 24%), and malignancy (*n* = 48, 23%) (Table 3).

**Table 3.** Underlying causes of anaemia of chronic disease

	Additional investigations, *n* (%) (*n* = 205; 77%)	Underlying disease already apparent, *n* (%) (*n* = 31; 12%)	No additional investigation, no apparent cause, *n* (%) (*n* = 31; 12%)	Total, *n* (%) (*n* = 267; 100%)
**Underlying cause established**	179 (87)	31 (100)	0 (0)	210 (79)
Autoimmune disease	41 (23)	10 (32)	–	51 (24)
Infection	68 (38)	–	–	68 (32)
Renal failure	4 (2)	–	–	4 (2)
Recent operation	2 (1)	1 (3)	–	3 (1)
Malignancy	44 (25)	4 (13)	–	48 (23)
Diabetes	7 (4)	12 (39)	–	19 (9)
Heart failure	5 (3)	2 (7)	–	7 (3)
Chronic lung disease	4 (2)	1 (3)	–	5 (2)
Other causes^a^	4 (2)	1 (3)	–	5 (2)
**No cause established**	26 (13)	0 (0)	31 (100)	57 (21)

Percentage of underlying causes is calculated from the total number of causes established in each subgroup. ^a^Such as liver cirrhosis, haematoma, and alcohol abuse.

In 154 patients (58%), the median time from presentation with anaemia to clarification of the underlying cause could be collected; this was 2 weeks (range 0–48). Of these 154 patients, the underlying cause was established within 1 week for 66 (43%), and within 4 weeks after presentation for 103 patients (67%). Further information is available from the authors on request.

### Current guideline recommendations

In 205 (77%) patients, the GP acted according to the current Dutch guidelines (additional investigations to clarify the underlying illness causing ACD [unless an underlying cause was already known], and no oral iron supplementation prescribed). These recommendations were adhered to more frequently in patients with a high ferritin level, that is >324 μg/l (OR 2.46; 95% confidence interval [CI] = 1.33 to 4.52), as shown in Table 4. In addition, less frequent implementation of the recommendations in older patients (OR 0.97, 95% CI = 0.94 to 1.00) and patients with more severe anaemia, that is haemoglobin <11.8 g/dl (OR 0.47, 95% CI = 0.2 to 1.03) was detected, but these associations were not statistically significant.

**Table 4.** Factors associated with implementation of the current recommendations and prescription of oral iron supplementation.

	Univariate analysis	Multivariate analysis
	Percentage or mean difference (95% CI)	Odds ratio (95% CI)
**Recommendations**		
Age, years	3.63 (0.65 to 6.60)	0.97 (0.94 to 1.00)
Female	84.0	Reference category
Male	91.9	0.82 (0.38 to 1.75)
Haemoglobin ≥11.8 g/dl	90.4	Reference category
Haemoglobin <11.8 g/dl	86.6	0.47 (0.21 to 1.03)
Ferritin >324 μg/l	92.5	Reference category
Ferritin ≤324 μg/l	84.3	2.46 (1.33 to 4.52)
**Oral iron supplementation**		
Age, years	-1.29 (-5.05 to 2.47)	1.00 (0.97 to 1.04)
Female	15.1	Reference category
Male	11.5	2.00 (0.83 to 4.83)
Haemoglobin ≥11.8 g/dl	6.4	Reference category
Haemoglobin <11.8 g/dl	19.0	4.97 (1.82 to 13.62)
Ferritin >324 μg/l	8.3	Reference category
Ferritin ≤324 μg/l	17.9	2.30 (1.06 to 4.99)

CI = confidence interval

### Oral iron supplementation

Of all 267 patients, 35 (13%) received oral iron supplementation despite being diagnosed with ACD and demonstrating ferritin levels ≥100 μg/l. Severe anaemia (haemoglobin <11.8 g/dl) and less enhanced ferritin levels (<324 μg/l) were associated with a higher probability of oral iron supplementation (OR 4.97; 95% CI = 1.82 to 13.62 and OR 2.30; 95% CI = 1.06 to 4.99, respectively), as shown in Table 4.

## Discussion

### Summary

This study provides a representative overview of the diagnostic strategies employed by GPs for their patients with ACD and an overview of the underlying causes as established by GPs and/or medical specialists. Additional investigations were requested in 77% of the analysed 267 ACD patients. The most frequently requested additional investigations were chest x-ray (24%), referral to an internist (21%), and referral to the emergency room (10%). The underlying cause of ACD was established in 79% of these patients, with the most common causes being infection (in 32%), autoimmune disease (in 24%), and malignancy (in 23%). The median time from presentation with anaemia to clarification of the underlying cause of ACD was 2 weeks. The prescription of oral iron supplementation in patients with ACD is not recommended and requires caution.^14–16^

### Strengths and limitations

A possible limitation of this study is related to the recruitment of patients. Of all GPs participating in the project, about one-third declined participation (mainly due to time constraints). However, the group of newly diagnosed ACD patients *not* included in this study presented no significant difference in sex, or haemoglobin, ferritin, or transferrin levels compared with those included in the analyses. In addition, because a difference in age and in serum iron levels was observed between the groups, all analyses were corrected for these two variables. Another limitation is the retrospective design of the study, which can lead to differences in the completeness of individual GPs’ medical reports and, perhaps, to loss of patient information, such as the reason to decline additional investigations after establishment of ACD.

Strengths of the study include a large sample size of representative patients providing a realistic overview of patients with ACD in general practice. In addition, patients were selected based on well-defined laboratory criteria, making these results generalisable to other countries. Finally, the content of the Dutch guidelines used for handling newly diagnosed patients with ACD is also widely accepted in other guidelines worldwide.^2,20,23^

### Comparison with existing literature

This study demonstrated that 77% of the ACD patients received additional investigations after establishment of the ACD, with the most common strategy being referral to a medical specialist (47%). This relatively large proportion of referrals might be because the diagnosis or treatment of the underlying cause of ACD might be beyond the scope of GPs’ focus, for example, the diagnosis or treatment of malignancies. In addition, for 12% of patients without a clear cause of ACD, no additional investigations were requested. For most of these patients, no reason was documented for disregarding further investigations. However, GPs may have considered these patients too vulnerable for extensive tests due to, for example, advanced age and/or comorbidities, or the lack of consequences in these frail patients.^24^

Comparable studies analysing the underlying causes of ACD have investigated hospitalised populations, whereas no data are available for community-dwelling adults with ACD. In this study, the most common underlying causes of ACD were infections, autoimmune inflammation, and malignancies; this is in line with data from hospitalised patients with ACD.^4,25,26^ However, compared with the present study population, a markedly higher prevalence of renal insufficiency was reported among hospitalised patients with ACD.^3,25^ This might be due to selection bias in the present study population; the transmural project separated renal anaemia as a distinct category of anemia, resulting in less renal insufficiency in this ACD patient group. In addition, since only patients with newly diagnosed anaemia (that is, no anaemia in the preceding 2 years) were included, this probably excluded patients frequently hospitalised and those already being treated by, for instance, a nephrologist.

In this study, the diagnostic strategy of GPs was in accordance with the current Dutch recommendations in 77% of the patients with newly diagnosed ACD. Patients presenting with high ferritin levels (>324 μg/l) were more often treated according to these recommendations. Since increased ferritin is a diagnostic parameter for ACD, elevated levels of ferritin might be an alarm symptom for GPs, prompting them to more frequently perform additional investigations. In addition, more increased ferritin levels might exclude iron deficiency more clearly, thereby preventing unnecessary oral iron supplementation. A trend was observed towards less frequent implementation of the recommendations in older patients and patients with more severe anaemia. In older patients, multiple diseases and/or comorbidities often determine the clinical presentation (that is, health status). Therefore, especially in these patients, GPs might decide to forgo additional investigations. Patients with severe anaemia may be rapidly treated with oral iron supplementation to elevate haemoglobin levels. However, this is not recommended in patients with ACD.

Nevertheless, about 13% of the patients in this study still received oral iron supplementation as part of their treatment, even though no benefit is expected due to enhanced levels of hepcidin and, therefore, reduced oral iron absorption.^2,14–16^ In this study, severe anaemia and minimally reduced ferritin levels (i.e. <324 μg/l) are associated with increased oral iron prescription by GPs. The association of minimally reduced ferritin levels and the more frequent prescription of oral iron supplementation might be caused by the absence of a definite ferritin cut-off value defining iron deficiency.^16,21^ The effect of oral iron supplementation in patients with ACD might cause unnecessary side effects and drug–drug interactions, and can lead to unnecessary (although small) costs.^27,28^

### Implications for practice

To the authors' knowledge, this is the first study to explore and describe additional investigations to establish underlying causes in patients with newly diagnosed ACD in primary care. In these patients, infections, autoimmune diseases, and malignancies were the most frequently occurring underlying causes. Oral iron supplementation, which is not indicated in ACD patients, needs to be abandoned in primary care to prevent unnecessary side effects and costs, as well as possible drug–drug interactions. The information emerging from this study and data on the prevalence of the underlying causes in primary care may lead to more targeted investigations and, potentially, to a more targeted diagnosis and more appropriate treatment.
